# Involvement of long non-coding RNA in colorectal cancer: From benchtop to bedside (Review)

**DOI:** 10.3892/ol.2015.2846

**Published:** 2015-01-05

**Authors:** LE-CHI YE, DE-XIANG ZHU, JUN-JUN QIU, JIANMIN XU, YE WEI

**Affiliations:** 1 Department of Oncological Surgery, The First Affiliated Hospital of Wenzhou Medical University, Wenzhou, P.R. China; 2 Department of General Surgery, Zhongshan Hospital, Fudan University, Shanghai, P.R. China; 3 Department of Gynecology, Obstetrics and Gynecology Hospital of Fudan University, Shanghai, P.R. China

**Keywords:** long non-coding RNA, colorectal cancer

## Abstract

Colorectal cancer (CRC) is one of the greatest threats to public health. Recent advances in whole-genome transcriptome analyses have enabled the identification of numerous members of a novel class of non-coding (nc)RNA, long ncRNA (lncRNA), which is broadly defined as RNA molecules that are >200 nt in length and lacking an open reading frame. In the present review, all lncRNAs associated with CRC are briefly summarized, with a particular focus on their potential roles as clinical biomarkers. CRC-associated lncRNAs involved in the underlying mechanisms of CRC progression are also initially included. This should benefit the development of novel markers and effective therapeutic targets for patients with CRC.

## 1. Introduction

Colorectal cancer (CRC) is currently the third most common malignancy worldwide (1). The development of CRC is a stepwise progression from benign polyps to invasive adenocarcinomas and distant metastases. Although advancements have been made with regard to the available treatment options, improvements in the survival rates of patients with CRC have been restricted due to the lack of early detection and optimal prognostic predictions, which require the exploration of corresponding biomarkers and an understanding of the molecular mechanisms of CRC.

Over the last 10 years, data from a number of high-throughput genomic platforms has indicated that the evolution of the developmental processes regulating complex organisms may be attributed to not only the protein-coding regions of the genome, but the non-coding regions as well (2). Non-coding RNAs (ncRNAs), which are transcribed from non-coding regions, lack an open reading frame and therefore have no apparent protein-coding capacity (3). Regulatory ncRNAs are classified empirically as small (18–200 nt) or long ncRNAs (lncRNAs; between 200 nt and >100 kb) based on the size of the functional RNA molecule (4).

In contrast to small ncRNAs, such as microRNAs (miRs), which have been extensively studied for their biological roles in cancer processes (5), lncRNAs are relatively less well described. However, the inherent biology of lncRNAs, often referred to as the dark matter of the genome, is gradually being elucidated (3). Previous studies have revealed that a large number of lncRNAs play significant roles in regulating cellular development and differentiation, processes that are frequently deregulated in cancer (6).

The present mini-review introduces all the CRC-associated lncRNAs known to date and concentrates on the potential utility of lncRNAs as diagnostic and prognostic tools in CRC. The aim of this review is to improve the understanding of the role of lncRNAs in CRC, which could lead to novel prevention strategies and early detection.

## 2. Classical lncRNAs associated with CRC

The H19-lncRNA is a paternally imprinted (maternally expressed) oncofetal gene that is abundantly expressed in a number of types of cancer. H19-derived miR-675 promotes human CRC cell growth and malignant transformation by targeting the tumor suppressor retinoblastoma protein (RB) (7). However, one study found that the depletion of H19 resulted in an increased polyp count in a mouse model of CRC (8). The cellular environment of the tumor type may determine this dual role as an oncogene and tumor suppressor. Although the hypermethylation of a differentially-methylated region (DMR) upstream of the H19 gene may result in activation of the normally silent maternal allele of the insulin-like growth factor-II gene (IGF2) (9), hypomethylation of the H19 DMR and a DMR upstream of IGF2, is observed in the CRC and normal mucosa of a single patient (10). This finding suggests that the favored IGF2-H19 enhancer competition model for IGF2 imprinting is not applicable in CRC. High H19 expression has been observed in liver metastases (LMs) derived from primary CRC alone (11). Ohana *et al* (12) and Sorin *et al* (13) developed vectors carrying the diphtheria toxin A (DTA) chain gene driven by H19 regulatory sequences and administered these plasmids intra-arterially in the CC531 rat colorectal LM (CLM) model. The results showed that the DTA-H19 plasmid significantly delayed tumor growth, indicating that lncRNA could be a therapeutic target in CRC.

A previous study reported that HOX transcript antisense RNA (HOTAIR) reprograms chromatin organization and promotes breast cancer metastasis (14). Pádua Alves *et al* (15) observed that the colon cancer stem cell subpopulation (CD133^+^/CD44^+^) exhibits higher HOTAIR levels compared with the non-stem cell subpopulation. This result indicates that the role of the HOTAIR-lncRNA in CRC progression is associated with the acquisition of stemness. HOTAIR expression levels were also found to be higher in CRC tissues compared with the corresponding normal tissues, and high HOTAIR expression correlated with the presence of LMs (16). Furthermore, patients with high expression levels of HOTAIR exhibited a relatively poor prognosis. Using cDNA array data, a gene set enrichment analysis of a subset of 32 CRC specimens revealed a close correlation between HOTAIR expression and members of polycomb repressive complex 2 (PRC2) (16). This finding suggested that HOTAIR expression is associated with a genome-wide reprogramming of PRC2 function in CRC.

Metastasis-associated lung adenocarcinoma transcript 1 (MALAT1)-lncRNA was first identified in metastatic non-small cell lung carcinoma (17), and its high expression was subsequently found to be associated with CRC metastasis (18). MALAT1 mutations occur in CRC cell lines or tissues, with one MALAT1 fragment (spanning nucleotides 6918–8441) being an important biological motif in metastasis (19). Furthermore, a study indicated that the downregulation of MALAT1 by resveratrol could decrease the nuclear localization of β-catenin and attenuate Wnt/β-catenin signaling, thereby inhibiting CRC invasion and metastasis (20). This study indicated the potential use of MALAT1 as a marker for early metastasis in patients with CRC.

Highly upregulated in liver cancer (HULC)-lncRNA was first identified in a screen for deregulated genes in a hepatocellular carcinoma-specific gene library (21). The upregulation of HULC was also detected in LMs from CRC, although no HULC was detected in the primary CRC samples (22). Furthermore, a lack of HCLC gene expression was found *in vitro* in the CRC HT-29 cell line, as well as in tumors induced by the direct administration of HT-29 cells into the liver of athymic mice over the course of two weeks (22). This finding may indicate that the liver microenvironment is responsible for increased HULC expression in CLM. Furthermore, in a pilot experiment, HULC was detected in peripheral blood cells obtained from healthy volunteers by reverse transcription-quantitative polymerase chain reaction (PCR) (23), indicating that lncRNA may be useful as a circulating biomarker in CRC.

As an imprinted gene, maternally-expressed gene 3 (MEG3) (24) is expressed in normal intestinal mucosa, whereas its expression is lost in CRC cells (including HT29 and HCT116 cells) (25). In culture and colony formation, the re-activation of MEG3 expression inhibits tumor cell proliferation. This inhibition of growth is partly a consequence of the apoptosis induced by MEG3. MEG3 induces p53 protein accumulation and stimulates transcription from a p53-dependent promoter (26). The aforementioned data suggests that MEG3-lncRNA functions as a tumor suppressor in CRC.

## 3. CRC-specific associated lncRNAs

CCAT1 (CRC-associated transcript 1) was identified in a study by Nissan *et al* (27), which reported its high expression in CRC, but not in normal tissues. Furthermore, this lncRNA was also found to be significantly upregulated in metastatic tissue. Analysis of RNA obtained from five patients with CRC metastases to either the liver or the peritoneal cavity revealed the >100-fold upregulation of CCAT1 compared with normal colon tissue, with four samples recorded with >450-fold upregulation. Significantly, CCAT1 overexpression was also reported in 40.0% of peripheral blood samples from CRC patients, but not from the samples of healthy controls (27). Thus, it has been suggested that testing for CCAT1 expression can detect small numbers of CRC cells. Additionally, a CCAT1-specific peptide nucleic acid-based molecular beacon was used to detect CRC (28), and the results showed CCAT1 expression in all (4/4) subjects with pre-cancerous adenomas and in all (8/8) patients with invasive CRC, which further proved that CCAT1 is a potential biomarker for early CRC diagnosis.

Recently, CCAT2, which encompasses the rs6983267 single nucleotide polymorphism (SNP), was also reported to be highly overexpressed in microsatellite-stable CRC, and to promote tumor growth and metastasis (29). This lncRNA may regulate Myc and Wnt in CRC pathogenesis and provide an alternative explanation for SNP-conferred cancer risk (29).

Colorectal neoplasia differentially expressed (CRNDE) is an lncRNA gene that is overexpressed in >90% of CRC tissues relative to paired healthy tissues (30). CRNDE expresses multiple splice variants, and the expression levels of CRNDE-h demonstrate a sensitivity of 95% and specificity of 96% for colon adenoma versus normal tissue. The study by Graham *et al* (30) showed that the level of CRNDE-h-lncRNA in plasma was positive for 87% of patients with CRC, but only 7% of healthy individuals. Thereafter, CRNDE was proven to be upregulated in gliomas, and its different splice forms are known to provide specific functional scaffolds for regulatory complexes (31). Recently, another study showed that CRNDE is regulated by insulin/IGFs and promotes the metabolic changes by which cancer cells evoke the Warburg effect (32), indicating CRNDE upregulation in CRC. Therefore, CRNDE may serve as an ideal biomarker for the early diagnosis of CRC.

In one recent study, low LOC285194-lncRNA expression was shown to be correlated with more distant metastasis in patients with CRC (P=0.046) (33), which indicated that this lncRNA plays a role as a tumor suppressor in the CLM process.

The overexpressed in colon carcinoma-1 (OCC-1) gene is transcribed as two regulatory lncRNAs. Elevated OCC-1-lncRNA levels were present in three out of eight CRC samples compared with the normal mucosa of the same patient, even though the same characteristics were present in each tumor (34). This data indicates that OCC-1-lncRNA overexpression may be a hallmark of CRC.

## 4. Other lncRNAs associated with CRC

Ultraconserved region transcripts (T-UCR) are a novel class of lncRNAs transcribed from UCRs (35,36). UCRs are frequently located in the intra- and intergenic regions (37), and aberrant T-UCR expression is also involved in CRC progression. The expression of uc.73A(P) was found to be significantly upregulated in CRC (37), as a decrease in its overexpression induced apoptosis and anti-proliferative effects in CRC cells abnormally expressing this T-UCR. However, the expression of uc.388 was reported to be significantly decreased in CRC samples and was associated with the distant metastasis of CRC and other effects (38). By screening genomic DNA for sequence variations in UCR loci in patients with CRC, Wojcik *et al* (39) found UCR mutations in CRC and created a catalog of DNA sequence variations in UCRs in human cancers. These findings indicated that further investigation of the genetic variations in ncRNAs may aid in the identification of additional biomarkers for CLM.

As one type of lncRNA, pseudogenes have long been labeled as ‘noise DNA’, inactive copies of genes that arise during genome evolution (40). However, recent results showed that pseudogene transcripts are often deregulated between cancer and normal tissue (41), indicating their involvement in tumor progression. The POU5F1 transcript, also known as octamer binding transcription factor 4, is believed to be one of the key regulators of cellular pluripotency (42). The POU5F1 pseudogene, POU5F1P1, is not only overexpressed in prostate cancer (43), but is also strongly associated with an increased risk of CRC. Furthermore, a genome-wide association study showed that the rs6983267 SNP in the POU5F1P1 region was significantly associated with decreased survival time in patients with stage III CRC (44). The tumor suppressor phosphatase and tensin homolog (PTEN) is significantly correlated with CRC (45), and its pseudogene, PTENP1 (also known as PTENpg1), can parallel PTEN and play a growth-suppressive role in CRC cells, although the PTENP1 locus is selectively lost in CRC (46,47). Myosin light chain kinase pseudogene 1 (MYLKP1) is partially duplicated from the original MYLK gene, which encodes non-muscle and smooth muscle myosin light chain kinase (smMLCK) isoforms (48). MYLKP1 overexpression can inhibit the expression of smMLCK in CRC cells by decreasing RNA stability, resulting in the increased proliferation of cells; accordingly, smMLCK is markedly decreased in CRC tissues compared with normal colon tissues (49). These studies suggest a novel biological role for pseudogene expression in CRC.

Plasmacytoma variant translocation 1 (PVT1), a >300-kb locus found downstream of c-Myc on chromosome 8q24 (50), produces a wide range of spliced lncRNAs. Compared with healthy tissues, PVT1-lncRNA is overexpressed in breast and ovarian cancer (51), indicating that PVT1 may be an oncogene. PVT1 small interfering RNA-transfected CRC cells exhibit a significant loss in their abilities of proliferation and invasion. Additionally, multivariate analysis found that the level of PVT1 expression was an independent risk factor for overall survival in patients with CRC (52). Unexpectedly, transcription of the PVT1 locus can be induced by p53 and consequently upregulates miR-1204, which inhibits HCT116 cell proliferation (53). Therefore, the precise interplay between miRs and other ncRNAs of the PVT1 locus within the context of the p53 pathway requires further exploration.

Loss of imprinting (LOI) of H19 genes in CRC is associated with CRC progression (10,54), and another lncRNA, long QT intronic transcript 1 (LIT1) (55), also called Kcnq1ot1 (56), also frequently exhibits LOI in CRC. Additionally, Nakano *et al* (57) further found that the LOI of LIT1 via epigenetic disruption plays an important role in CRC.

Recently, Zhai *et al* (58) identified that long intergenic ncRNA (lincRNA)-p21 expression is significantly higher in patients with stage III tumors compared with those with stage I tumors, and that elevated lincRNA-p21 levels are significantly associated with a higher degree of vascular invasion. However, another study (59) showed decreases in lincRNA-p21 in CRC tissues. Furthermore, the enforced expression of lincRNA-p21 enhances sensitivity to radiotherapy in CRC by promoting apoptosis, the reason for which may be suppression of the β-catenin signaling pathway. The Warburg effect is known to play an important role in CRC progression, yet it remains unclear whether lncRNAs are involved in this phenomenon. Yang *et al* (60) first showed that lincRNA-p21 is a hypoxia-responsive lncRNA that is essential for hypoxia-enhanced glycolysis, which suggested the involvement of lincRNA-p21 in the regulation of the Warburg effect.

ncRNA expressed in aggressive neuroblastoma (ncRAN)-lncRNA was first recognized in a chromosomal gain, behaving as an oncogene in aggressive neuroblastomas (61). Thereafter, Qi *et al* (62) proved that the *in vitro* migration and invasion of CRC cells is mediated by ncRAN, suggesting that this lncRNA may be a novel prognostic indicator and biomarker for the early diagnosis of CRC. Recently, a novel lncRNA, prostate cancer associated-transcript 1 (PCAT1), was identified to be highly overexpressed in aggressive prostate cancer (63). In addition, PCAT1 was also revealed to be overexpressed in CRC tissues compared with matched normal tissues, and there was a significant association between higher PCAT1 expression and distant metastasis and poor overall survival (64). Notably, a search of the University of California Santa Cruz Human Genome Browser database (Feb 2009 assembly) (65) revealed that one adjoining neighbor of PCAT1 on chromosome 8q24, named prostate cancer-associated non-coding RNA 1 (PRNCR1; also known as PCAT8) (66) is also correlated with CRC. Li *et al* (67) conducted a case-control study and genotyped five tag SNPs in PRNCR1 in 313 patients with CRC and 595 control subjects using a PCR-restriction fragment length polymorphism assay. The results showed that rs13252298 and rs1456315 were associated with significantly decreased risks of CRC, indicating that SNPs in PRNCR1-lncRNA may contribute to the susceptibility to CRC. Low expression in tumor (LET)-lncRNA was reported to be downregulated in CRC as a regulator of hypoxia signaling, offering novel avenues for therapeutic intervention against the progression of cancer (68).

## 5. Challenges and future perspectives

**Figure 1:**
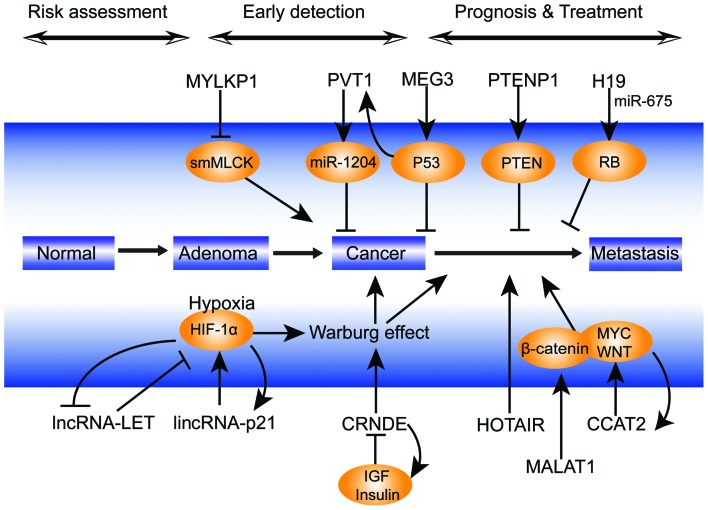
Potential mechanism of lncRNAs involved in colorectal cancer progression. lncRNA, long non-coding RNA; lincRNA, long intergenic non-coding RNA; miR, microRNA; HIF, hypoxia inducible factor; IGF, insulin-like growth factor; PTEN, phosphatase and tensin homolog; PTENP1, PTEN pseudogene 1; RB, retinoblastoma protein; smMLCK, smooth muscle myosin light chain kinase; HOTAIR, HOX transcript antisense RNA; MALAT1, metastasis-associated lung adenocarcinoma transcript 1; CRNDE, colorectal neoplasia differentially expressed; MYLKP1, myosin light chain kinase pseudogene 1; CCAT2, colorectal cancer-associated transcript 2; PVT1, plasmacytoma variant translocation 1; MEG3, maternally-expressed gene 3; LET, low expression in tumor.

From a clinical perspective, a large number of dysregulated ncRNAs represent a number of useful biomarkers for the diagnosis and prognosis of patients with CRC (Table I). However, a series of challenges remain to be addressed. Firstly, there is a considerable lack of understanding with regard to the regulation of lncRNA expression and the detailed mechanisms (Fig. 1) involved in lncRNA-mediated effects on tumor progression. Therefore, it may be an over-simplification to classify all tumor-associated lncRNAs into CRC activator or suppressor genes. Secondly, the problems associated with the variability in lncRNA detection in CRC samples should be overcome using standardized techniques for tissue isolation and preparation, platforms and software analysis to avoid selection bias. Thirdly, a number of prognostic or predictive biomarkers for CRC that are based on *in vitro* observation fail when they are translated into clinical management. To succeed in the future, further validation of these potential lncRNA biomarkers in additional cohorts (particularly in different ethnic groups) or prospective randomized trials is required to aid in the measurement of the true effect of these lncRNAs and their possible roles in CRC treatment. Recent studies have also suggested the possibility of a widespread interaction network involving competing endogenous RNAs, whereby lncRNAs modulate regulatory RNAs by binding to and titrating them away from their mRNA-binding sites (69,70). In addition to providing the possibility of an additional level of post-transcriptional regulation, such a network also necessitates the reassessment of the existing regulatory pathways involved in CRC progression and metastasis.

In conclusion, evidence is accumulating that lncRNAs have a significant role in the CRC process and may serve as potential CRC biomarkers for diagnosis and prognosis. However, further lncRNAs involved in the metastasis of CRC remain to be identified. Therefore, continued investigation is necessary to yield additional information on CRC-associated lncRNAs for future use in clinical practice.

## Figures and Tables

**Table I:** CRC-associated lncRNAs.

lncRNA	Potential mechanism	Expression	Function	Locus	Size (kb)	Reference
H19	Control of imprinting	Upregulated	⇅↓ proliferation?	Chr11p15.5	2.3	(7,11,12)
HOTAIR	Gene silencing by binding to PRC2 and LSD1	Upregulated	↑metastasis ↑poor prognosis	Chr12q13.13	2.2	(15,16)
MALAT1	RNA splicing; small RNA production; protein interaction	Upregulated	↑invasion ↑metastasis	Chr11q13.1	~7	(19)
HULC	RNA-DNA (CREB)	Upregulated	N.D.	Chr6p24.3	0.5	(22)
MEG3	Increases p53 levels by suppressing MDM2 levels	Downregulated	↓proliferation ↑apoptosis	Chr14q32	1.6–1.8	(25)
CCAT1	Unknown	Upregulated	↑risk of CRC	Chr8q24.21	2.6	(27,28)
CCAT2	Regulates Myc and Wnt	Upregulated	↑proliferation ↑metastasis	Chr8q24	0.4	(29)
CRNDE	Provides scaffolds for regulatory complexes	Upregulated	↑Warburg effect ↑risk of CRC	Chr16:hCG_1815491	~10	(30)
LOC285194	Unknown	Downregulated	↑metastasis	Chr3q13.31	2.1	(33)
OCC-1	Unknown	Upregulated	N.D.	Chr12q24.1	1.2–1.3	(34)
lincRNA-p21	Binds to hnRNP; guides it to p53-targeted gene promoters	Up- or downregulated	↑invasion ↑radiation sensitivity ↑Warburg effect	Upstream of p21/Cdkn1a	~3.1	(60)
UC.388	Unknown	Downregulated	↓metastasis	Near BX641000/TCF12/FLJ14957 genes	0.2–0.8	(37,38)
UC.73A	Unknown	Upregulated	↑proliferation ↓apoptosis	Near AK126774, BC017741, ZFHX1B	0.2	(37,38)
LIT1 (Kcnq1ot1)	LOI	LOI occurs frequently	N.D.	Chr11p15.5	91	(57)
PTENP1	Pseudogene of PTEN	Downregulated	↓proliferation	Chr9q13.3	~3.9	(47)
MYLKP1	Pseudogene of MYLK	Upregulated	↑proliferation	Chr3p12.3	106	(49)
pou5f1p1 (OCT4)	Pseudogene of pou5f1	Upregulated	↑risk of CRC	Chr8q24	0.4	(44)
UCA1	Unknown	Upregulated	N.D.	Chr19p13.12	1.4, 2.2, 2.7	(71)
PCAT1	Inhibits BRCA2	Upregulated	↑proliferation ↑poor prognosis	Chr8q24	1.9	(64)
PRNCR1	Unknown	Upregulated	↑proliferation	Chr8q24	13	(66,67)
LET	Regulator of hypoxia signaling	Downregulated	↓metastasis	Chr15q24.1	2.3	(68)
ncRAN		Upregulated	↑migration ↑invasion	Chr17q25.1	2.3	(62)
PVT1	A p53-inducible target miR-1204	Upregulated	↓apoptosis ↑invasion ↑poor prognosis	Chr8q24.21	>300	(52)

CLM, colorectal liver metastasis; CRC, colorectal cancer; N.D., not determined; lncRNA, long non-coding RNA; lincRNA, long intergenic non-coding RNA; miRNA, microRNA; LOI, loss of imprinting; HOTAIR, HOX transcript antisense RNA; MALAT1, metastasis-associated lung adenocarcinoma transcript 1; CRNDE, colorectal neoplasia differentially expressed; MYLKP1, myosin light chain kinase pseudogene 1; CREB, cAMP response element-binding protein; hnRNP, heterogeneous ribonucleoprotein particle; PRC2, polycomb repressive complex 2; LSD1, lysine-specific demethylase 1; PCAT1, prostate cancer-associated transcript 1; PRNCR1, prostate cancer-associated non-coding RNA 1; PTENP1, phosphatase and tensin homolog 1; UCA1, urothelial cancer-associated 1; CCAT, CRC-associated transcript; HULC, highly upregulated in liver cancer; OCC-1, overexpressed in colon carcinoma-1; PVT1, plasmacytoma variant translocation 1; ncRAN, non-coding RNA expressed in aggressive neuroblastoma; MEG3, maternally-expressed gene 3; L1T1, long QT intronic transcript 1; LET, low expression in tumor.

## References

[1] Jemal A, Bray F, Center MM, Ferlay J, Ward E, Forman D (2011). Global cancer statistics. CA Cancer J Clin.

[2] Esteller M (2011). Non-coding RNAs in human disease. Nat Rev Genet.

[3] Ponting CP, Oliver PL, Reik W (2009). Evolution and functions of long noncoding RNAs. Cell.

[4] Kapranov P, Cheng J, Dike S (2007). RNA maps reveal new RNA classes and a possible function for pervasive transcription. Science.

[5] Bartel DP (2004). MicroRNAs: genomics, biogenesis, mechanism, and function. Cell.

[6] Gutschner T, Diederichs S (2012). The hallmarks of cancer: a long non-coding RNA point of view. RNA Biol.

[7] Tsang WP, Ng EK, Ng SS (2010). Oncofetal H19-derived miR-675 regulates tumor suppressor RB in human colorectal cancer. Carcinogenesis.

[8] Yoshimizu T, Miroglio A, Ripoche MA (2008). The H19 locus acts in vivo as a tumor suppressor. Proc Natl Acad Sci USA.

[9] Moulton T, Crenshaw T, Hao Y (1994). Epigenetic lesions at the H19 locus in Wilms’ tumour patients. Nat Genet.

[10] Cui H, Onyango P, Brandenburg S, Wu Y, Hsieh CL, Feinberg AP (2002). Loss of imprinting in colorectal cancer linked to hypomethylation of H19 and IGF2. Cancer Res.

[11] Fellig Y, Ariel I, Ohana P (2005). H19 expression in hepatic metastases from a range of human carcinomas. J Clin Pathol.

[12] Ohana P, Schachter P, Ayesh B (2005). Regulatory sequences of H19 and IGF2 genes in DNA-based therapy of colorectal rat liver metastases. J Gene Med.

[13] Sorin V, Ohana P, Mizrahi A (2011). Regional therapy with DTA-H19 vector suppresses growth of colon adenocarcinoma metastases in the rat liver. Int J Oncol.

[14] Gupta RA, Shah N, Wang KC (2010). Long non-coding RNA HOTAIR reprograms chromatin state to promote cancer metastasis. Nature.

[15] Pádua Alves C, Fonseca AS, Muys BR (2013). Brief report: The lincRNA Hotair is required for epithelial-to-mesenchymal transition and stemness maintenance of cancer cell lines. Stem cells.

[16] Kogo R, Shimamura T, Mimori K (2011). Long noncoding RNA HOTAIR regulates polycomb-dependent chromatin modification and is associated with poor prognosis in colorectal cancers. Cancer Res.

[17] Gutschner T, Hämmerle M, Eissmann M (2013). The noncoding RNA MALAT1 is a critical regulator of the metastasis phenotype of lung cancer cells. Cancer Res.

[18] Chang JL, Li ZG, Wang XY, Yang MH (2008). Detection of p53, MALAT1, ki-67 and β-catenin mRNA expression and its significance in molecular diagnosis of colorectal carcinoma. World Chinese J Digestol.

[19] Xu C, Yang M, Tian J, Wang X, Li Z (2011). MALAT-1: a long non-coding RNA and its important 3′ end functional motif in colorectal cancer metastasis. Int J Oncol.

[20] Ji Q, Liu X, Fu X (2013). Resveratrol inhibits invasion and metastasis of colorectal cancer cells via MALAT1 mediated Wnt/β-catenin signal pathway. PLoS One.

[21] Panzitt K, Tschernatsch MM, Guelly C (2007). Characterization of HULC, a novel gene with striking up-regulation in hepatocellular carcinoma, as noncoding RNA. Gastroenterology.

[22] Matouk IJ, Abbasi I, Hochberg A, Galun E, Dweik H, Akkawi M (2009). Highly upregulated in liver cancer noncoding RNA is overexpressed in hepatic colorectal metastasis. Eur J Gastroenterol Hepatol.

[23] Xie H, Ma H, Zhou D (2013). Plasma HULC as a promising novel biomarker for the detection of hepatocellular carcinoma. Biomed Res Int.

[24] Miyoshi N, Wagatsuma H, Wakana S (2000). Identification of an imprinted gene, Meg3/Gtl2 and its human homologue MEG3, first mapped on mouse distal chromosome 12 and human chromosome 14q. Genes Cells.

[25] Zhang X, Zhou Y, Mehta KR (2003). A pituitary-derived MEG3 isoform functions as a growth suppressor in tumor cells. J Clin Endocrinol Metab.

[26] Zhou Y, Zhang X, Klibanski A (2012). MEG3 noncoding RNA: a tumor suppressor. J Mol Endocrinol.

[27] Nissan A, Stojadinovic A, Mitrani-Rosenbaum S (2012). Colon cancer associated transcript-1: a novel RNA expressed in malignant and pre-malignant human tissues. Int J Cancer.

[28] Kam Y, Rubinstein A, Naik S (2014). Detection of a long non-coding RNA (CCAT1) in living cells and human adenocarcinoma of colon tissues using FIT-PNA molecular beacons. Cancer Lett.

[29] Ling H, Spizzo R, Atlasi Y (2013). CCAT2, a novel noncoding RNA mapping to 8q24, underlies metastatic progression and chromosomal instability in colon cancer. Genome Res.

[30] Graham LD, Pedersen SK, Brown GS (2011). Colorectal neoplasia differentially expressed (CRNDE), a novel gene with elevated expression in colorectal adenomas and adenocarcinomas. Genes Cancer.

[31] Ellis BC, Molloy PL, Graham LD (2012). CRNDE: A long non-coding RNA Involved in CanceR, Neurobiology, and DEvelopment. Front Genet.

[32] Ellis BC, Graham LD, Molloy PL (2014). CRNDE, a long non-coding RNA responsive to insulin/IGF signaling, regulates genes involved in central metabolism. Biochim Biophys Acta.

[33] Liu Q, Huang J, Zhou N (2013). LncRNA loc285194 is a p53-regulated tumor suppressor. Nucleic Acids Res.

[34] Pibouin L, Villaudy J, Ferbus D (2002). Cloning of the mRNA of overexpression in colon carcinoma-1: a sequence overexpressed in a subset of colon carcinomas. Cancer Genet Cytogenet.

[35] Peng JC, Shen J, Ran ZH (2013). Transcribed ultraconserved region in human cancers. RNA Biol.

[36] Scaruffi P (2011). The transcribed-ultraconserved regions: a novel class of long noncoding RNAs involved in cancer susceptibility. ScientificWorldJournal.

[37] Calin GA, Liu CG, Ferracin M (2007). Ultraconserved regions encoding ncRNAs are altered in human leukemias and carcinomas. Cancer Cell.

[38] Sana J, Hankeova S, Svoboda M, Kiss I, Vyzula R, Slaby O (2012). Expression levels of transcribed ultraconserved regions uc.73 and uc388 are altered in colorectal cancer. Oncology.

[39] Wojcik SE, Rossi S, Shimizu M (2010). Non-coding RNA sequence variations in human chronic lymphocytic leukemia and colorectal cancer. Carcinogenesis.

[40] Ng SY, Gunning P, Eddy R (1985). Evolution of the functional human beta-actin gene and its multi-pseudogene family: conservation of noncoding regions and chromosomal dispersion of pseudogenes. Mol Cell Biol.

[41] Poliseno L (2012). Pseudogenes: newly discovered players in human cancer. Sci Signal.

[42] Wezel F, Pearson J, Kirkwood LA, Southgate J (2013). Differential expression of Oct4 variants and pseudogenes in normal urothelium and urothelial cancer. Am J Pathol.

[43] Kastler S, Honold L, Luedeke M (2010). POU5F1P1, a putative cancer susceptibility gene, is overexpressed in prostatic carcinoma. Prostate.

[44] Panagopoulos I, Möller E, Collin A, Mertens F (2008). The POU5F1P1 pseudogene encodes a putative protein similar to POU5F1 isoform 1. Oncol Rep.

[45] Ali A, Saluja SS, Hajela K, Mishra PK, Rizvi MA (2014). Mutational and expressional analyses of PTEN gene in colorectal cancer from Northern India. Mol Carcinog.

[46] Johnsson P, Ackley A, Vidarsdottir L (2013). A pseudogene long-noncoding-RNA network regulates PTEN transcription and translation in human cells. Nat Struct Mol Biol.

[47] Poliseno L, Salmena L, Zhang J, Carver B, Haveman WJ, Pandolfi PP (2010). A coding-independent function of gene and pseudogene mRNAs regulates tumour biology. Nature.

[48] Lazar V, Garcia JG (1999). A single human myosin light chain kinase gene (MLCK; MYLK). Genomics.

[49] Han YJ, Ma SF, Yourek G, Park YD, Garcia JG (2011). A transcribed pseudogene of MYLK promotes cell proliferation. FASEB J.

[50] Rack KA, Delabesse E, Radford-Weiss I (1998). Simultaneous detection of MYC, BVR1, and PVT1 translocations in lymphoid malignancies by fluorescence in situ hybridization. Genes Chromosomes Cancer.

[51] Guan Y, Kuo WL, Stilwell JL (2007). Amplification of PVT1 contributes to the pathophysiology of ovarian and breast cancer. Clin Cancer Res.

[52] Takahashi Y, Sawada G, Kurashige J (2014). Amplification of PVT-1 is involved in poor prognosis via apoptosis inhibition in colorectal cancers. Br J Cancer.

[53] Barsotti AM, Beckerman R, Laptenko O, Huppi K, Caplen NJ, Prives C (2012). p53-Dependent induction of PVT1 and miR-1204. J Biol Chem.

[54] Nakagawa H, Chadwick RB, Peltomaki P, Plass C, Nakamura Y, de La Chapelle A (2001). Loss of imprinting of the insulin-like growth factor II gene occurs by biallelic methylation in a core region of H19-associated CTCF-binding sites in colorectal cancer. Proc Natl Acad Sci USA.

[55] Murakami K, Oshimura M, Kugoh H (2007). Suggestive evidence for chromosomal localization of non-coding RNA from imprinted LIT1. J Hum Genet.

[56] Mitsuya K, Meguro M, Lee MP (1999). LIT1, an imprinted antisense RNA in the human KvLQT1 locus identified by screening for differentially expressed transcripts using monochromosomal hybrids. Hum Mol Genet.

[57] Nakano S, Murakami K, Meguro M (2006). Expression profile of LIT1/KCNQ1OT1 and epigenetic status at the KvDMR1 in colorectal cancers. Cancer Sci.

[58] Zhai H, Fesler A, Schee K, Fodstad O, Flatmark K, Ju J (2013). Clinical significance of long intergenic noncoding RNA-p21 in colorectal cancer. Clin Colorectal Cancer.

[59] Wang G, Li Z, Zhao Q (2014). LincRNA-p21 enhances the sensitivity of radiotherapy for human colorectal cancer by targeting the Wnt/β-catenin signaling pathway. Oncol Rep.

[60] Yang F, Zhang H, Mei Y, Wu M (2014). Reciprocal regulation of HIF-1α and lincRNA-p21 modulates the Warburg effect. Mol Cell.

[61] Yu M, Ohira M, Li Y (2009). High expression of ncRAN, a novel non-coding RNA mapped to chromosome 17q25.1, is associated with poor prognosis in neuroblastoma. Int J Oncol.

[62] Qi P, Xu MD, Ni SJ (2014). Down-regulation of ncRAN, a long non-coding RNA, contributes to colorectal cancer cell migration and invasion and predicts poor overall survival for colorectal cancer patients. Mol Carcinog.

[63] Yang L, Lin C, Jin C (2013). lncRNA-dependent mechanisms of androgen-receptor-regulated gene activation programs. Nature.

[64] Ge X, Chen Y, Liao X (2013). Overexpression of long noncoding RNA PCAT-1 is a novel biomarker of poor prognosis in patients with colorectal cancer. Med Oncol.

[65] Meyer LR, Zweig AS, Hinrichs AS (2013). The UCSC Genome Browser database: extensions and updates 2013. Nucleic Acids Res.

[66] Chung S, Nakagawa H, Uemura M (2011). Association of a novel long non-coding RNA in 8q24 with prostate cancer susceptibility. Cancer Sci.

[67] Li L, Sun R, Liang Y (2013). Association between polymorphisms in long non-coding RNA PRNCR1 in 8q24 and risk of colorectal cancer. J Exp Clin Cancer Res.

[68] Yang F, Huo XS, Yuan SX (2013). Repression of the long noncoding RNA-LET by histone deacetylase 3 contributes to hypoxia-mediated metastasis. Mol Cell.

[69] Salmena L, Poliseno L, Tay Y, Kats L, Pandolfi PP (2011). A ceRNA hypothesis: the Rosetta Stone of a hidden RNA language?. Cell.

[70] Tay Y, Rinn J, Pandolfi PP (2014). The multilayered complexity of ceRNA crosstalk and competition. Nature.

[71] Wang F, Li X, Xie X, Zhao L, Chen W (2008). UCA1, a non-protein-coding RNA up-regulated in bladder carcinoma and embryo, influencing cell growth and promoting invasion. FEBS Lett.

